# Perspectives on the Role of Fospropofol in the Monitored Anesthesia Care Setting

**DOI:** 10.1155/2011/458920

**Published:** 2011-04-14

**Authors:** Joseph V. Pergolizzi, Tong J. Gan, Stanford Plavin, Sumedha Labhsetwar, Robert Taylor

**Affiliations:** ^1^Department of Medicine, Johns Hopkins University School of Medicine, Baltimore, MD 21205-2196, USA; ^2^Department of Anesthesiology, Georgetown University School of Medicine, Washington, DC 20057, USA; ^3^NEMA Research Inc., Naples, FL 34108-1877, USA; ^4^Duke University Medical Center, Durham, NC 27710, USA; ^5^Ambulatory Anesthesia of Atlanta, Atlanta, GA 30328, USA

## Abstract

Monitored anesthesia care (MAC) is a safe, effective, and appropriate form of anesthesia for many minor surgical procedures. The proliferation of outpatient procedures has heightened interest in MAC sedation agents. Among the most commonly used MAC sedation agents today are benzodiazepines, including midazolam, and propofol. Recently approved in the United States is fospropofol, a prodrug of propofol which hydrolyzes in the body by alkaline phosphatase to liberate propofol. Propofol liberated from fospropofol has unique pharmacological properties, but recently retracted pharmacokinetic (PK) and pharmacodynamic (PD) evaluations make it difficult to formulate clear conclusions with respect to fospropofol's PK/PD properties. In safety and efficacy clinical studies, fospropofol demonstrated dose-dependent sedation with good rates of success at doses of 6.5 mg/kg along with good levels of patient and physician acceptance. Fospropofol has been associated with less pain at injection site than propofol. The most commonly reported side effects with fospropofol are paresthesia and pruritus. Fospropofol is a promising new sedation agent that appears to be well suited for MAC sedation, but further studies are needed to better understand its PK/PD properties as well its appropriate clinical role in outpatient procedures.

## 1. Introduction

One of the most profound changes in clinical practice in the past two decades has been the migration of many procedures from the inpatient to the outpatient setting. In 2006, about two-thirds of all diagnostic and therapeutic procedures in the United States were performed on an outpatient basis [1], and procedures performed in freestanding ambulatory care centers have increased 300% from 1996 to 2006 [2]. The trend toward outpatient surgery has accelerated interest in alternatives to general anesthesia (GA), including monitored anesthesia care (MAC). 

Propofol is a commonly used agent in MAC and other clinical settings, but supplies of this agent are low, owing to recalls and a possible decision by one manufacturer to no longer make the drug [3]. Lean manufacturing techniques can produce cost effective products, but such a supply chain can be particular vulnerable to disruptions such as voluntary recalls and market shifts [4]. Thus, a drug of choice for MAC sedation may be temporarily unavailable. Propofol has also suffered from recent media attention on its potential for misuse [5]. When a familiar agent is suddenly in short supply, there may be potential safety risks to patients even if an alternative agent(s) is available, in that clinicians may not be familiar with the other agents, which may have a different mechanism of action, safety profile, or other properties [6]. 

Fospropofol, a prodrug of propofol, might be considered the “logical” alternative to propofol, but its potential role in MAC sedation may have been affected by a series of pharmacokinetic studies [7–11] that had to be retracted from the literature [12]. The current and potential role of fospropofol in the MAC setting remains unclear.

## 2. Monitored Anesthesia Care

MAC is not to be confused with *moderate sedation*, sometimes called *conscious sedation*, defined by the American Society of Anesthesiologists (ASA) as sedation during which the patient can be aroused by verbal commands or light touch and in which a patent airway and stable cardiac and respiratory functions can be maintained [13]. Moderate sedation is not adequate for many procedures, including some outpatient operations.

MAC has been defined by the ASA as the specific service of an anesthesiologist to a patient undergoing a diagnostic or therapeutic procedure [14]. MAC is considered the standard of care for patients undergoing a wide range of procedures, such as minor surgeries [15], and is an appropriate choice for delivery of anesthesia in many outpatient procedures [16]. During MAC, the anesthesiologist or a member of the anesthesia care team provides a number of specific services, including, but not limited to, the following.

- Assessment and management of the patient's actual or anticipated physiological derangements or medical problems that may occur during a diagnostic or therapeutic procedure.
- Ability to convert to GA when necessary, due to the likelihood that deep sedation may intentionally or unintentionally transition to GA.
- Ability to intervene in order to rescue the patient's airway from any sedation-induced compromise.
- Readiness with acute clinical interventions and possible resuscitation in the event that medications precipitate adverse physiologic responses.
- Monitoring of vital signs, maintenance of the patient's airway and continuous evaluation of vital functions.
- Diagnosis and treatment of clinical problems that occur during the procedure.
- Administration of sedatives, analgesics, hypnotics, anesthetic agents, or other medications, as necessary, to ensure patient safety and comfort.
- Postprocedural responsibilities, including assuring a full return to consciousness, pain relief, management of adverse physiological responses or side effects from medications administered during the procedure, as well as diagnosis and treatment of coexisting medical problems.

While morbidity and mortality were once the main criteria by which to judge an anesthetic agent, today cost effectiveness and patient satisfaction must be taken into account, although they can be difficult to measure [17]. Patient dissatisfaction has been associated with measurable variables such as longer time in the operating room [18] and postoperative symptoms in the first 24 hours following surgery [19], whereas improved patient satisfaction has been associated with subjective variables such as patient-perceived concern and courtesy exhibited by the clinical team [20, 21]. Cost effectiveness is often assessed together with patient satisfaction [22, 23], but their interrelationship poses its own challenges [24]. 

Early cost effectiveness studies of propofol found relative acquisition costs of propofol expensive [25–27] but these studies were done before a generic product was available. Compared to sevoflurane, propofol (*n* = 104) exhibited an improved recovery profile, significantly higher levels of patient satisfaction, and *overall lower costs *[28]. However, cost effectiveness studies sometimes evaluate only acquisition or procedural costs and do not factor in the expenses of managing side effects such as postoperative nausea and vomiting (PONV) or postdischarge nausea and vomiting (PDNV), which may increase the patient's total cost of care [29]. PONV/PDNV is one of the most common reasons for outpatients to be admitted to the hospital, occurring in about 1% of patients [30]. The rates of PONV/PDNV vary substantially with several risk factors, including type of anesthesia, type of procedure, and sex [31]. The incidence of PONV is lower with propofol than sevoflurane (an inhalational agent used in general anesthesia), but this may be an apples-to-oranges comparison [32]. While PONV/PDNV following ambulatory procedures is an important consideration, these conditions have not been quantified in the literature for fospropofol.

Current propofol labeling requires its administration by an anesthesiologist [33] which may be argued to add to its relative cost. While one economic model for the use of propofol in GI procedures found propofol more cost effective than meperidine and midazolam, even when accounting for extra personnel [34], the costs for an anesthesiology provider have been reported to add between $250 and $400 to the cost of a colonoscopy [35]. For that and other reasons, the FDA has been asked to approve propofol administration by nonanesthesiologists in certain limited settings [35]. To bolster this point of view, a report in the literature of global data from 646,080 cases found mortality rates associated with endoscopist-directed propofol sedation were similar to mortality rates of GA administered by anesthesiologists and superior to the published mortality rates for endoscopist-administered opioids and benzodiazepines [36]. There remains considerable controversy as to whether and under what conditions propofol should ever be administered by nonanesthesiologists [37–41]. A computer-aided propofol sedation system (Sedasys Computer-Assisted Personalized Sedation System, Ethicon Endo-Surgery, Johnson & Johnson Company, New Brunswick, NJ) has been designed to allow gastroenterologists and nurses to administer propofol sedation in specific outpatient procedures without benefit of an anesthesiologist by continuously monitoring and recording such parameters as oxygen saturation, respiratory rate, heart rate, blood pressure and end-tidal carbon dioxide [42]. This system, which is approved in the European Union for certain gastrointestinal (GI) procedures, was rejected by the FDA in April 2010 [43]. 

Fospropofol disodium has recently been approved in the United States for use in the MAC sedation in adults undergoing diagnostic or therapeutic procedures (Lusedra, Eisai, Research Triangle, NC) [44] and clinical trial data have reported it to be effective and generally well tolerated [45]. It is a water-soluble phosphate ester prodrug of propofol [46], available in a single-dose vial for injection (35 mg/ml), requiring no infusion equipment. From a technological aspect, fospropofol is easier to administer than propofol, which may heighten the controversy as to whether or under what circumstances fospropofol might be appropriately used in MAC procedures by nonanesthesiologists. The safety of fospropofol (Lusedra) for continuous sedation has not been established and therefore its use is not recommended [47]. Fospropofol was administered to 38 intubated and mechanically ventilated patients in postoperative and intensive care settings. An occurrence of nonsustained ventricular tachycardia was observed as a serious adverse reaction in one patient in the study. Another patient with acute myeloid leukemia with renal and hepatic insufficiency experienced a further increase in plasma format concentration from a baseline of 66 mcg/mL to a postdose level of 212 mcg/mL after a 12-hour infusion [47]. The clinical significance of these findings is unknown.

## 3. MAC Agents

The ideal sedation agent for MAC procedures would be safe, efficacious, cost effective, convenient, with predictable pharmacological properties and few side effects. It should also be well accepted by both patients and their physicians. Among the most commonly used agents in MAC sedation are benzodiazepines, in particular diazepam and midazolam, and propofol. Benzodiazepines have been discussed in the literature for use in outpatient colonoscopy procedures [48], but may have slow or potentially variable onsets of action and prolonged periods of effect [16]. Of midazolam, diazepam, and propofol, midazolam was associated with the longest sedation and recovery times [49]. 

Propofol is an ultrashort-acting sedative hypnotic [50], which releases aminobutyric acid in the brain [16]. It is a phenolic derivative unrelated to other sedative/hypnotic agents [51]. Propofol is available only in an oil-in-water emulsion formulation, which readily crosses the blood-brain barrier because of its highly lipophylic nature [52]. It has a rapid onset of sedation, the level of which increases in a dose-dependent fashion. Propofol is associated with injection-site pain (incidence 32% to 67% for a single bolus) [53] and carries a slight but potentially lethal risk of bacterial infection [54]. 

In a comparative study of outpatient colonoscopy patients, those who received propofol achieved a significantly greater mean level of sedation than similar patients with midazolam/meperidine [55]. However, deeper sedation can impair the patient's cooperation during surgery (such as turning) and may necessitate rescue to more moderate levels of sedation. 

Propofol's narrow therapeutic index, lack of therapeutic antagonist [56], and lipid formulation [57] limit its use in some patient populations.

**Figure 1:**
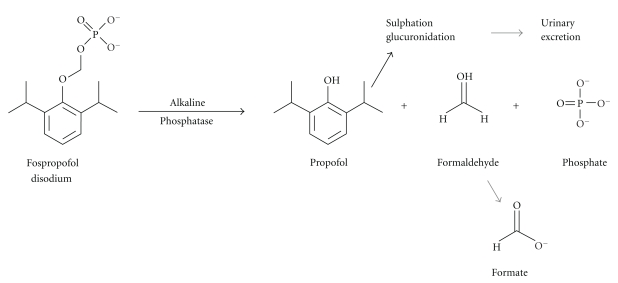
Formaldehyde converting to aldehyde dehydrogenase and then to formic acid (formate).

Fospropofol disodium (2,6-diisopropylphenoxymethyl phosphate, disodium salt/C_13_H_19_O_5_PNa_2_) is a novel prodrug, that is, a 2,6-dissoprophyl phenol molecule with a methyl phosphate group substituted at the first carbon hydroxyl on the base benzene structure. Using a charged phosphate group to replace a noncharged hydroxyl group introduces electronegativity and allows fospropofol to dissolve readily in water. Such hydrophilic additions have a long history of use in certain antibiotics and steroid drugs [58]. In simple terms, fospropofol undergoes hydrolysis by alkaline phosphatase in the endothelial cell surface, which causes it to release the active metabolite propofol, formaldehyde (which converts to formate) and phosphate [41, 59]. It is thought that the liberated propofol increases activity of gamma-aminobutyric acid (GABA) [60], the chief inhibitory neurotransmitters of the central nervous system (CNS), binding to GABA receptors, and, in this way, potentiating GABA-inhibitory synaptic currents [61]. See Figure 1.

A number of agents can be used for MAC sedation, but shortages of propofol have focused interest in fospropofol. While the literature reports a great deal about midazolam, benzodiazepam, and propofol, less is known about the newer agent fospropofol. 

## 4. Pharmacokinetics and Pharmacodynamics of Fospropofol

The recent retraction [12] of six studies of the pharmacokinetic (PK) and pharmacodynamic (PD) properties of fospropofol [7–11, 62] due to possible errors in propofol assays have clouded discussion about the PK/PD of fospropofol [63]. According to investigators, all six initial studies on the pharmacokinetic and pharmacodynamic properties of fospropofol and its tolerability had been published when an analytical propofol assay inaccuracy was discovered [64]. All six studies were phase I or phase II studies sponsored by Guilford Pharma (Baltimore, MD). Guilford Pharma later became MGI Pharma (Baltimore, MD). The studies were conducted in two independent academic facilities in Europe (Gent, Belgium and Erlangen, Germany). The sponsor developed and validated a specific propofol assay and the original publications were authored by both academic and industry experts. When the error was detected, MGI Pharma stated that it intended within 12 months to conduct further studies, publish those results, and then using the new results estimate the degree of error from the previously published studies [63]. The matter was complicated when the ownership of the drug was transferred in the middle of 2009 from MGI Pharma to Eisai (Woodcliff Lake, NJ). The original investigators were unable to conduct new studies within the 12-month deadline and thus requested the studies to be retracted because the error and possibly the conclusions reached in this studies were flawed [64]. 

It is known that the PK characteristics of propofol liberated from fospropofol differ from the PK of propofol emulsion administered intravenously [65] with lower peak concentrations and more prolonged plasma concentrations [66]. 

**Figure 2:**
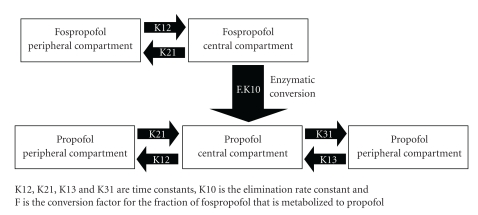
The five-compartment model of fospropofol liberating propofol [67].

A randomized double-blind phase III study of 18 colonoscopy patients pretreated with fentanyl randomized to 6.5 mg/kg or 2 mg/kg fospropofol with midazolam as a reference helped to develop the five-compartment PK model of fospropofol [67]. This model shows fospropofol as a two-compartment agent which liberates propofol, a three-compartment model. See Figure 2. 

The enzymatic process which liberates propofol from fospropofol occurs within a 15 to 20-minute time window [68], which creates a slower onset of action and, thus, a different sedation profile than propofol emulsion. The recommended *maximum dose* of fospropofol is 12.5 mg/kg, which should lead to loss of consciousness in about four minutes [16]. The recommended effective dose is 6.5 mg/kg. Fospropofol plasma concentrations were predictive of effect-site concentrations. 

## 5. Efficacy of Fospropofol in MAC Sedation

There are few published studies on clinical efficacy of fospropofol in MAC sedation. The authors reviewed four clinical studies of fospropofol to evaluate its efficacy, summarized in Table 1.

Fospropofol resulted in significant dose-dependent increases in sedation success from 24% (2 mg/kg), 35% (5 mg/kg), 69% (6.5 mg/kg) to 96% (8 mg/kg) (*P* < .001) in a randomized, double-blind clinical trial of 127 adult patients undergoing sedation for a colonoscopy [69]. Patients were pretreated with 50 *μ*g of fentanyl five minutes prior to the initial dose of sedative and then received either fospropofol (2, 5, 6.5 or 8 mg/kg) or midazolam (0.02 mg/kg). Supplemental medication was permitted if needed to reach a modified observer's assessment of alertness/sedation (MOAA/S) of ≤4. The majority of fospropofol patients (all groups) had mean MOAA/S scores ranging from 2 to 4 during the colonoscopy. Midazolam, a frequently employed anesthetic agent in colonoscopy, was used as a reference but not as a direct comparator. Sedation success and patient satisfaction were higher among the fospropofol groups than midazolam patients. Patients in the 6.5 mg/kg fospropofol group reported higher satisfaction with fospropofol than the other fospropofol dose groups. Patients reporting being satisfied with fospropofol were, by percentage 72.0% for 2.0 mg/kg; 84% for 5.0 mg/kg; 92.3% for 6.5 mg/kg; and 79.2% for 8.0 mg/kg. Patient satisfaction in the midazolam group was 69.2%. Physician satisfaction increased with dosage of fospropofol (8.0% for 2.0 mg/kg; 11.5% for 5.0 mg/kg; 26.9% for 6.5 mg/kg; and 50.0% for 8.0 mg/kg; physician satisfaction with midazolam was 11.5%). When patients and physicians were asked if they would use this sedative again, the greatest percentage in agreement occurred in the fospropofol 6.5 mg/kg group (96.2% for patients, 92.3% of physicians). Physicians were significantly more likely to say they would use fospropofol again in the 6.5 mg/kg group than the 8.0 mg/kg group (*P* < .001). 

A study by Cohen and colleagues compared 6.5 mg/kg of fospropofol to 2.0 mg/kg in 314 patients undergoing colonoscopy with midazolam 0.02 mg/kg used as a reference [70]. Patients were pretreated with fentanyl 50 *μ*c. Those patients who received 6.5 mg/kg of fospropofol had significantly greater sedation success (*P* < .001), that is 87% for 6.5 mg/kg versus 26% for 2.0 mg/kg (midazolam was 69%) and were significantly less likely to remember being awake during the procedure (*P* < .001), having recall rates of 51% for 6.5 mg/kg versus 100% for 2.0 mg/kg (midazolam 60%). Memory retention was comparable for both fospropofol doses (70% and 82%, resp.) and 41% for midazolam. Physician satisfaction was significantly higher for 6.5 mg/kg of fospropofol (*P* < .001) compared to 2.0 mg/kg. 

Silvestri et al reported on the safety and efficacy of fospropofol in a phase III randomized, double-blind study of flexible bronchoscopy patients (*n* = 252) [71]. Following pretreatment with fentanyl 50 *μ*g, patients were randomized to receive fospropofol 2 mg/kg or 6.5 mg/kg. Supplemental doses were given per protocol and the endpoint of the study was sedation success as evaluated by three consecutive MOAA/S scores of ≤4 plus procedure completion without the need of alternative sedative or mechanical ventilation. Sedation success rates were 88.7% (6.5 mg/kg) and 27.5% (2 mg/kg) with significantly more patients sedated successfully at the 6.5 mg/kg dose (*P* < .001). Patients given the 6.5 mg/kg were significantly more likely say they would use that anesthesia again (*P* < .001) and were significantly more likely to not recall the procedure (*P* < .001). Patients dosed with 6.5 mg/kg of fospropofol were significantly less likely to require alternative sedation (*P* < .001). The median time to full alertness was 5.5 min (6.5 mg/kg) versus 3.0 min (2 mg/kg). 

The Silvestri study is of particular interest because 41% of patients enrolled were geriatric (≥65 years old) and 43% had an ASA Physical Classification System status of 3 or 4, that is, there was significant comorbidity. 

Rex and colleagues reported on a double-blind, randomized, multicenter study of 314 patients undergoing colonoscopy; in this study, patients were first pretreated with fentanyl 50 *μ*g and then received either 2.0 or 6.5 mg/kg of fospropofol or 0.02 mg/kg of midazolam as a reference [72]. The 87% rate of sedation success in the 6.5 mg/kg fospropofol group was significantly greater (*P* < .001) than the 26% achieved with 2.0 mg/kg fospropofol (69% midazolam). Furthermore, the mean time from first dose to sedation was significantly briefer (*P* < .0001) for 6.5 mg/kg fospropofol compared to 2.0 mg/kg fospropofol (8.6 ± 5 min versus 16.6 ± 5 min, resp.). The time until patients were fully alert was comparable in both fospropofol groups: 6.7 ± 7.5 min for 6.5 mg/kg fospropofol compared to 8.9 ± 8.4 min for 2.0 mg/kg fospropofol (findings not statistically significant). The time until patients could be discharged was similar (and not significantly different) in both groups: 8.7 ± 7.6 min for 6.5 mg/kg fospropofol versus 8.9 ± 8.9 min for 2.0 mg/kg fospropofol. There was a significant difference in the depth of sedation. Patients who received 6.5 mg/kg fospropofol had MOAA/S scores of 2 to 4 from the first dose of study medication to fully alert status 63.8% (±20.2) compared to 47.2% (±20.9) among patients who received 2.0 mg/kg fospropofol. The depth of sedation was significantly greater for 6.5 mg/kg than 2.0 mg/kg fospropofol patients (*P* < .001). 

**Figure 3:**
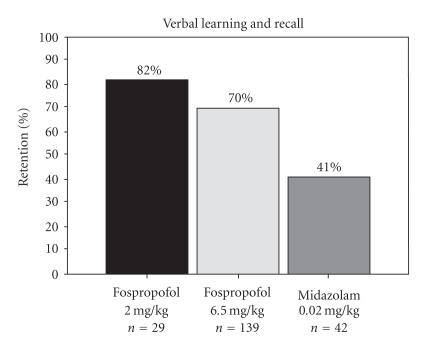
Learning and recall in fospropofol patients (2.0 mg/kg versus 6.5 mg/kg) with midazolam as reference.

Rex and colleagues also evaluated sedation success and clear-headed recovery in the same multicenter, double-blind phase III clinical trial (*n* = 314) of patients undergoing colonoscopy [73]. Cognitive testing at baseline (Hopkins Verbal Learning Test-Revised) was performed to evaluate recall and memory. Patients were randomized to receive fospropofol (2.0 mg/kg), fospropofol (6.5 mg/kg) or midazolam (0.02 mg/kg). At baseline, mean retention percentages for verbal learning and recall were similar in all three groups, but fospropofol groups (2 mg/kg and 6.5 mg/kg) had significantly higher mean percentage of retention postprocedure than the midazolam patients (*P* < .001), (see Figure 3). This showed that patients receiving 6.5 mg/kg fospropofol had high rates of sedation success with better memory retention than patients given midazolam.

These studies of fospropofol used midazolam rather than propofol as comparator, but for clinical practice, it may be more relevant to compare propofol to fospropofol. Fospropofol is associated with less injection site pain than propofol [11] and significantly less respiratory depression [41, 74]. The latter may make fospropofol particularly useful in treating patients with comorbidities or at risk for respiratory events.

## 6. Onset of Action

Predictable onset of action is crucial to a MAC sedation agent, but both rapid and slow onset of action present distinct attributes that may be clinically useful. A MAC sedation agent with a rapid onset of action allows for more immediate and possibly more comfortable sedation. Propofol, for example, has a rapid onset of action, which can be advantageous in the outpatient setting, where patient throughput and patient satisfaction are particularly emphasized. 

Fospropofol is generally considered to have a slow onset of action of from four to 13 minutes compared to propofol, which has a rapid onset of action of about 40 seconds [75]. This slower onset of action can be attributed to the fact that fospropofol is a prodrug which must first metabolize in order to release propofol. This slower onset of action may make it possible, in some instances, to offer fewer boluses of medication for a short operation, possibly making fospropofol practical for use in an outpatient clinic performing very brief diagnostic or therapeutic procedures. The slow onset of action may require consideration in dosing, recognizing that there is a “lag time” between infusion and effect.

## 7. Safety of Fospropofol in MAC Sedation

Paresthesia is one of the most commonly reported adverse events with fospropofol sedation [46, 68, 69]. In the 2010 study by Cohen, paresthesia was reported to occur at a rate of 68% in the 6.5 mg/kg fospropofol group versus 60% in the 2.0 mg/kg group [70]. Pruritus occurred in 16% of the 6.5 mg/kg fospropofol group versus 26% of 2.0 mg/kg [70]. It has been observed that other medications containing phosphate esters (such as dexamethasone) have provoked similar side effects [76]. Hypoxia was also observed with fospropofol anesthesia, which investigators reported as mild and comparable to rates observed with midazolam and fentanyl. This hypoxia was resolvable with increased oxygen flow [71]. 

Fospropofol injections are associated with less pain than propofol injections [11] and are well tolerated [7]. 

The safety and tolerability of intravenous fospropofol for MAC sedation in the outpatient setting was evaluated in a recent study (*n* = 123) of patients undergoing minor surgery who were ASA P1 to P4 [77]. All patients were pretreated with fentanyl 50 *μ*g and then received 6.5 mg/kg IV fospropofol and up to five supplemental doses (1.63 mg/kg) until an MOAA/S score of ≤4 was achieved to maintain adequate sedation. Sedation depth, need for supplemental doses, use of alternative sedatives, and the nature of treatment-emergent and sedative-related adverse events were measured. The study found that 60% of patients could achieve adequate sedation to initiate and complete the procedure with two supplemental doses or fewer. About 5% required alternative sedatives. Following the procedure, 61% of patients were fully alert (MOAA/S = 5) two minutes after the procedure. The most common treatment-emergent adverse events were paresthesia (63%) and pruritus (28%). Adverse event profiles were similar in patients with hepatic disease (*n* = 20) or severe renal dysfunction (*n* = 5). The investigators of this study concluded that fospropofol had an acceptable safety and tolerability profile when used at an initial dose of 6.5 mg/kg.

## 8. Risks

In late 2009, the Deputy Administrator of the Drug Enforcement Administration (DEA) of the United States made fospropofol a schedule IV controlled substance [78]. Schedule IV drugs have a relatively low potential for abuse; Schedule IV includes benzodiazepines and certain long-acting barbiturates. While propofol has recently been in world news because of its association with the death of a prominent musician [79], propofol is not a controlled substance, although there have been recommendations that it be controlled by the Drug Enforcement Administration [5]. There is currently no abuse data for fospropofol, but it was determined that fospropofol could lead to physical or psychological dependence, based on what is known about propofol [78]. It has been speculated that fospropofol may possess a greater degree of “likability” compared to propofol by those who seek to misuse such drugs. 

## 9. Conclusion

The expansion of outpatient procedures has raised interest in MAC sedation and appropriate MAC sedation agents. While propofol is a frequently used agent in MAC sedation, it may be subject to shortages that force focus on other agents. Its prodrug fospropofol has been introduced to the market as a potential MAC sedation agent, offering a slower onset of action and requiring no special infusion equipment. Limited clinical experience and publications involving the use of fospropofol coupled with having six fundamental PK/PD fospropofol studies retracted have undermined the clinical community's ability to thoroughly assess this new drug and its potential role in MAC sedation. Published safety and efficacy studies of colonoscopies and bronchoscopies have clinical value, but are limited in that they report on the use of fospropofol in relatively short procedures, rather than longer procedures where repeated dosing might be required. More studies using fospropofol in lengthier procedures are needed. Furthermore, PK/PD studies of fospropofol are also needed to clarify the pharmacological properties of the agent. In terms of technical delivery, fospropofol may be suitable to administration by nonanesthesiologists because it requires no special infusion equipment, but whether or not this is clinically appropriate remains controversial and falls outside the scope of this paper. Fospropofol is currently labeled to be administered by anesthesiologists only. 

When administered in an intravenous bolus, fospropofol liberates propofol in the body, but this propofol has a different PK/PD profile from the propofol of the propofol emulsion. As such, fospropofol possesses a different sedation profile than propofol. In particular, fospropofol has a slower onset of action, which may confer advantages (less bolusing in short procedures) or disadvantages (possibly reduced patient comfort and more time spent in achieving adequate sedation). The most commonly reported side effects are paresthesia and pruritus. Fospropofol overcomes the side effect of injection site pain common for propofol. 

Fospropofol appears to be a promising new agent for MAC sedation but further studies are warranted to better evaluate its PK/PD properties and its appropriate role in the outpatient setting. In light of current potential shortfalls of propofol, such studies are urgently needed.

## Figures and Tables

**Table 1:** Key clinical trials evaluating the use of fospropofol as an agent in MAC sedation.

Study	*n*	Procedure	Pretreatment	Dose (mg/kg) of fospropofol	Other agents?	Conclusions
Cohen 2008 [69]	127	Colonoscopy	50 *μ*g fentanyl	2, 5, 6.5, or 8.0	Midazolam as reference (0.02 mg/kg)	Significant dose-dependent increases in sedation

Cohen et al. 2010 [70]	314	Colonoscopy	50 *μ*g fentanyl	2.0 or 6.5	Midazolam (0.02 mg/kg)	Significantly greater sedation success, greater memory retention, and higher physician satisfaction at 6.5 than 2.0 mg/kg of fospropofol

Silvestri et al. 2009 [71]	252	Flexible bronchoscopy	50 *μ*g fentanyl	2 or 6.5	No	Significantly higher sedation success at 6.5 mg/kg (88.7% versus 27.5%, *P* < .001)

Rex et al. 2007, Safety and Efficacy [72]	314	Colonoscopy	50 *μ*g fentanyl	2.0 or 6.5	Midazolam (0.02 mg/kg)	Significantly higher sedation success at 6.5 mg/kg (87% versus 26%, *P* < .001) with significantly greater depth of sedation

Rex et al. 2007, Clear-headed recovery [73]	314	Colonoscopy	50 *μ*g fentanyl	2.0 or 6.5	Midazolam (0.02 mg/kg)	Fospropofol patients (both doses) had significantly higher mean percentage of retention postprocedure than midazolam (*P* < .001)
